# The hygroscopic behavior of plant fibers: a review

**DOI:** 10.3389/fchem.2013.00043

**Published:** 2014-01-24

**Authors:** Amandine Célino, Sylvain Fréour, Frédéric Jacquemin, Pascal Casari

**Affiliations:** Institut de Recherche en Génie Civil et Mécanique (UMR CNRS 6183), Université de Nantes - Centrale Nantes, I.U.T. de Saint-NazaireSaint-Nazaire cedex, France

**Keywords:** natural fibers, composite materials, hydrophilic behaviors, ageing effects, durability

## Abstract

Environmental concern has resulted in a renewed interest in bio-based materials. Among them, plant fibers are perceived as an environmentally friendly substitute to glass fibers for the reinforcement of composites, particularly in automotive engineering. Due to their wide availability, low cost, low density, high-specific mechanical properties, and eco-friendly image, they are increasingly being employed as reinforcements in polymer matrix composites. Indeed, their complex microstructure as a composite material makes plant fiber a really interesting and challenging subject to study. Research subjects about such fibers are abundant because there are always some issues to prevent their use at large scale (poor adhesion, variability, low thermal resistance, hydrophilic behavior). The choice of natural fibers rather than glass fibers as filler yields a change of the final properties of the composite. One of the most relevant differences between the two kinds of fiber is their response to humidity. Actually, glass fibers are considered as hydrophobic whereas plant fibers have a pronounced hydrophilic behavior. Composite materials are often submitted to variable climatic conditions during their lifetime, including unsteady hygroscopic conditions. However, in humid conditions, strong hydrophilic behavior of such reinforcing fibers leads to high level of moisture absorption in wet environments. This results in the structural modification of the fibers and an evolution of their mechanical properties together with the composites in which they are fitted in. Thereby, the understanding of these moisture absorption mechanisms as well as the influence of water on the final properties of these fibers and their composites is of great interest to get a better control of such new biomaterials. This is the topic of this review paper.

## Introduction

Environmental concern has resulted in a renewed interest in bio-based materials. Among them, plant fibers are perceived as an environmentally friendly substitute to glass fibers for the reinforcement of composites, particularly in automotive engineering (Wambua et al., 2003; Suddell and Evans, 2005; Summerscales et al., 2010). Due to their wide availability, low cost, low density, high-specific mechanical properties, and eco-friendly image, they are increasingly being employed as reinforcements in polymer matrix composites (Bledzki and Gassan, 1999). In literature the term biocomposite is often used to define a polymeric matrix reinforced by natural fibers. The increasing number of publications during last 10 years including reviews, reflect the growing importance of these new biocomposites (Bledzki and Gassan, 1999; Mohanty et al., 2000; John and Thomas, 2008; Satyanarayana et al., 2009; Summerscales et al., 2010; Faruk et al., 2012). Indeed, their complex microstructure as a composite material makes plant fiber a really interesting and challenging subject to study. Research subjects about such fibers are abundant because there are always some issues to prevent their use at large scale (poor adhesion, variability, low thermal resistance, hydrophilic behavior). The choice of natural fibers rather than glass fibers as filler yields a change of the final properties of the composite. One of the most relevant differences between the two kinds of fiber is their response to humidity. Actually, glass fibers are considered as hydrophobic whereas plant fibers have a pronounced hydrophilic behavior. Composite materials are often submitted to variable climatic conditions during their lifetime, including unsteady hygroscopic conditions. However, in humid conditions, strong hydrophilic behavior of such reinforcing fibers leads to high level of moisture absorption in wet environments (Célino et al., 2013). This results in the structural modification of the fibers and an evolution of their mechanical properties together with the composites in which they are fitted in Dhakal et al. (2007); Symington et al. (2009); Placet et al. (2012b). Thereby, the understanding of these moisture absorption mechanisms as well as the influence of water on the final properties of these fibers and their composites is of great interest to get a better control of such new biomaterials. This is the topic of this review paper.

## About plant fibers

### Origin of plant fibers

**Figure 1:**
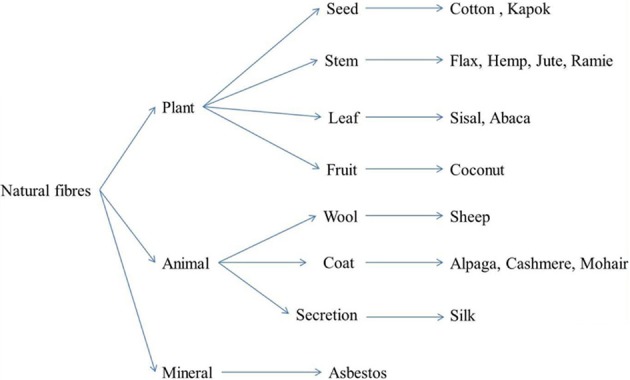
**Classification of natural fibers [inspired by Baley (2004)]**.

In nature, there is a wide range of natural fibers which can be distinguished by their origin. Precisely, natural fibers are divided into three categories including animal fibers, mineral fibers, and plant fibers (Figure 1). In the present paper we will focus on this last group. For details about the other kind of fibers, interesting readers could refer to (Speil and Leineweber, 1969; Champness et al., 1976; Blicblau et al., 1997; Fu et al., 2009). As can be seen in Figure 1, plant fibers could also be classified according to their location in the plant. For example, bast fibers as flax, hemp or jute (Mohanty and Misra, 1995; Summerscales et al., 2010) are extracted from the stem of the plant whereas other fibers could be extracted from seeds (cotton) (Chand et al., 1988), fruit (coconut, pineapple), (Arib et al., 2004) or even the leaves of the plant (sisal) (Mukherjee and Satyanarayana, 1984; Li et al., 2000). The origins and properties of these different fibers have been described in a detailed review paper by (Faruk et al., 2012). Vegetal fibers are extracted from the plant using widely known and controlled processes in the textile industry. Some authors studied the influence of growth conditions and extraction processes on their properties (Keller et al., 2001; Coroller et al., 2013; Martin et al., 2013). As an example in Martin et al. (2013) works, moisture sorption and mechanical properties of flax fibers were found to be dependent upon the degree of retting.

### Chemical and structural organization

#### Chemical composition

Plant fibers are mainly composed by sugar based polymers (cellulose, hemicelluloses) combined with lignin and pectin. Additional components, such as wax or oil could be found as well as structural water (De Rosa et al., 2010). Climatic conditions, age or degradation process influence the chemical composition which varies from plant to plant and within different part of a same plant. In their literature review (Faruk et al., 2012) listed the range of the average chemical constituent for a wide variety of plant type.

Cellulose is the major constituent of such fibers. It is a linear polymer chain consisting of D-glucopyranose units joined together by β-1,4-glycosidic linkages. Hydrogen bonds between the different macromolecules give the assembly various interesting physical properties, including the ability to form crystalline structures. As a result, cellulose has a semi crystalline form: there are both highly crystalline regions and amorphous regions. Crystalline cellulose displays six different polymorphs with the possibility of conversion from one form to another. The cellulose I crystal form, or native cellulose, also comprises two allomorphs, namely cellulose Iα and Iβ (Sugiyama et al., 1991). The ratio of these allomorphs is found to vary from plant to plant. In bast fibers as flax, jute or hemp, cellulose Iβ is found to be predominant (Sarko and Muggli, 1974; Nishiyama et al., 2002). Crystalline regions are called crystallites. The threadlike entity which arises from the linear association of these components is called the microfibril; it forms the basic structural unit of the plant cell wall. These microfibrils are composed by several thousands of cellulose chains. Their diameter can be measured by X-ray diffraction. It is in the nanometer range, between 5 and 30 nm, depending on the authors and the type of fiber (Näslund et al., 1988; Fink et al., 1990; Eichhorn et al., 2001; Astley and Donald, 2003). In the longitudinal direction, the Young's modulus of these microfibrils is about 137 GPa (Sakurada et al., 1962). These features provide their good mechanical properties to plant fibers. In most natural fibers, the microfibrils orient themselves at an angle to the fiber axis called the microfibril angle. This angle has a significant influence on the mechanical properties of the fiber. The lower it is, better the properties are (Bourmaud et al., 2013). Cave developed a technique to measure it by using X-ray diffraction (Cave, 1997). It varies from plant to plant. Resources on cellulose can be found at references (Eichhorn et al., 2001; Heinze and Fischer, 2005).

#### Structural organization

**Figure 2:**
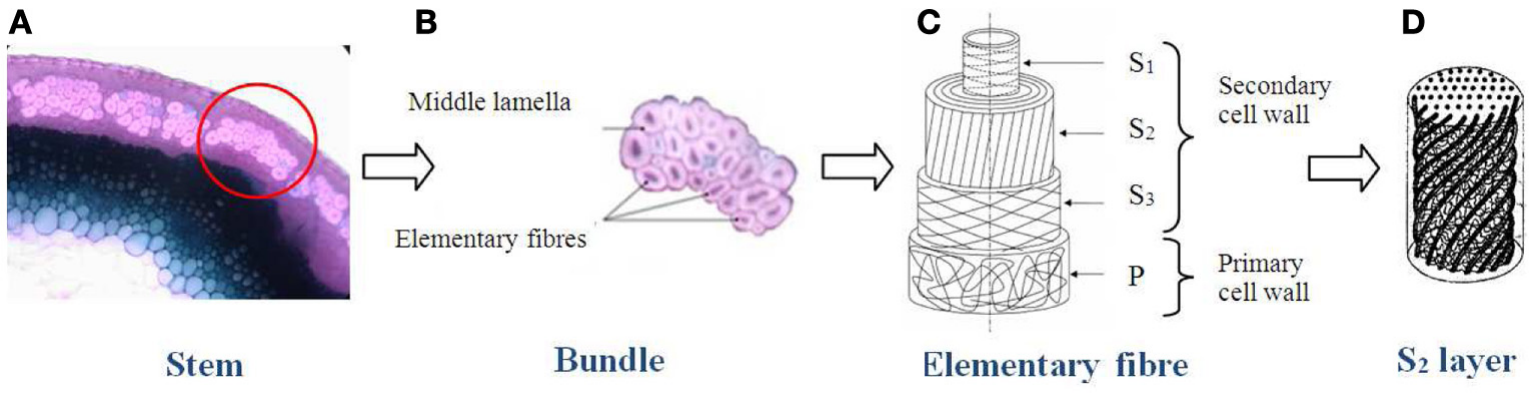
**Multi-scale structure of the flax fiber [Célino et al. (2013, 2014a,b) inspired by Baley (2002) and Morvan et al. (2003)]**. **(A)** Stem of a flax plant, **(B)** bundle of flax fibres, **(C)** represntation of an elementary fibre, **(D)** the S2 layer of elementary fibres.

Plant fibers have a multi-scale structure and they can be used at different scales for composite materials reinforcement (Figure 2). Indeed, fibers could be conditioned as fabric yarn (Madsen and Lilholt, 2003), bundle of fiber or even unit fibers (Baley, 2002; Placet, 2009). A bundle of fibers (Figure 2B), is a gathering of several elementary fibers, linked together by a ten micrometers wall mainly composed of pectin and lignin. This wall is called middle lamella (Morvan et al., 2003). Plant fiber yarns consist of a large number of relatively short plant fibers that are twisted with an angle to the yarn axis in order to provide axial strength to the yarn (Madsen et al., 2007). The unit fibers have a multi cell wall structure (Figure 2C). The section is polygonal but usually assumed as circular for the calculation of mechanical properties. Basically, it is represented by a hollow polyhedron, decomposed into several walls and layers. The external wall is called the primary wall. It presents a relatively small thickness compared to the total thickness of the fiber. This wall is essentially composed of pectin, low crystalline cellulose, hemicellulose and waxes in a lower amount (Gorshkova et al., 2000; Zykwinska et al., 2008). The secondary wall, which represents about 90% of the total section, is divided into three layers. It is mainly composed of cellulose microfibrils oriented parallel to each other and embedded in an amorphous matrix composed of hemicellulose, pectin, and lignin. The three layers are different from each other because of their different thickness and structural organization (microfibril angle, chemical composition). The thickest layer is the S2 layer. It represents 70–80% of the secondary wall thickness. Thus, the fiber properties are largely governed by the feature of this layer.

In first approximation, the unit fibers can be considered as composite materials with an amorphous matrix of hemicellulose and reinforced by cellulose microfibrils which are oriented in parallel and form a helix angle with the axis of the fiber. In the S2 layer, this angle is about 10° (Figure 2D). In the other layers, the microfibrils are not oriented at the same angles as shown in the schematic representation of Baley (2002). The hollowed part is called the lumen. It gives the fibers a tubular structure.

### Physical and mechanical properties

As mentioned before, plant fibers have the properties to compete with glass fibers as reinforcement for composite materials. Because of their low density, they have good specific mechanical properties, particularly concerning their stiffness. Table 1 presents the important mechanical properties of commonly used fibers (Oksman et al., 2003; Satyanarayana and Wypych, 2007; Bodros and Baley, 2008; Ochi, 2008; Summerscales et al., 2010; Bourmaud, 2011; Faruk et al., 2012).

**Table 1:** **Mechanical properties of different fibers (Oksman et al., 2003; Bodros and Baley, 2008; Ochi, 2008; Satyanarayana et al., 2009; Summerscales et al., 2010; Bourmaud, 2011; Faruk et al., 2012)**.

**Fiber**	**Density**	**Young's modulus (GPa)**	**Tensile strength (MPa)**	**Elongation at break (%)**
Flax	1.54	27.5–85	345–2000	1–4
Ramie	1.5–1.56	27–128	400–1000	1.2–3.8
Hemp	1.47	17–70	368–800	1.6
Jute	1.44	10–30	393–773	1.5–1.8
Sisal	1.45–1.5	9–22	350–700	2–7
Coconut	1.15	4–6	131–175	15–40
Cotton	1.5–1.6	5.5–12.6	287–597	7–8
Nettle	1.51	24.5–87	560–1600	2.1–2.5
Kenaf	1.2	14–53	240–930	1.6
Bamboo	0.6–1.1	11–17	140–230	–
E-glass	2.5	70	2000–3500	2.5
Carbone	1.4	230–240	4000	1.4–1.8

As seen in Table 1, the properties of plant fibers may differ for a given fiber. In fact, the major problem, with such fiber is the high variability of their properties. Thus, in literature, there is a large amount of data showing relatively wide distribution. First, this variability can be explained by differences in the fibers chemical composition and structure (microfibrillar angle, crystallinity, defects) due to the environmental conditions during the growth (Bourmaud et al., 2013). Secondly, it can be explained by different testing methods employed or different environmental conditions (relative humidity, temperature, speed loading, number of sample tested) (Placet et al., 2012a). Moreover, as mentioned before, plant fibers can be investigated at different scales (fiber bundles or unit fibers). In the literature, there are mechanical data from both fibers bundles (Madsen et al., 2007; Charlet et al., 2011) and elementary fibers (Baley, 2002; Placet et al., 2012b). When testing is performed at the bundle scale, there are slippage effects of the fibers relative to each other in the middle lamella. Thus, generally, the properties of fiber bundles are lower than those of the elementary fibers. Charlet et al. (2011) studied the mechanical behavior of the middle lamella of flax fiber bundles. Authors showed low shear strength of this interface which can explain the weaker mechanical properties of bundle.

As mentioned in Structural organization, a plant fiber is a composite made of three polymers (cellulose, hemicellulose, and lignin), in which the unidirectional cellulose microfibrils constitute the reinforcing elements in the matrix blend of hemicellulose and lignin. This structure could be built as multi-ply construction with layers P, S1, S2, and S3 of cellulose microfibrils presenting different angles to the fiber axis (Figure 2C). Then the elastic properties could be calculated using classical laminated theory (Gassan et al., 2001). Transition scales models should also be used to predict hygro-elastic properties of such fibers. Mori and Tanaka models for example should be developed as described in Fréour et al. (2006). In order to take into account the disorientation of microfibrils reinforcement, methods presented by Lacoste et al. (2010, 2011) should be used. Mechanical properties of these three polymers have been widely studied in literature. Tashiro and Kobayashi (1991) and Gillis (1969) for example, presented data about cellulose. Moreover, Cousins, in the 80s, helped to build a valuable database for the lignin and hemicellulose properties (Cousins et al., 1975; Cousins, 1976, 1978). Because of its specific structure, plant fibers have an anisotropic behavior. In the longitudinal direction, they have good mechanical properties via microfibrils whereas in the radial direction mechanical properties are lower and more variable because of the amorphous blend properties.

**Figure 3:**
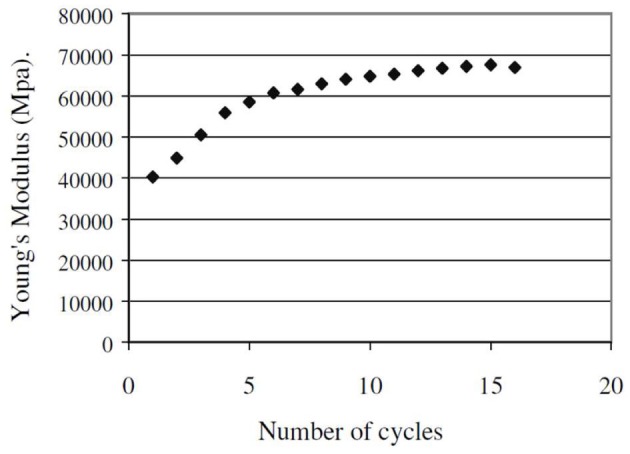
**Young's modulus evolution of flax fibers vs. number of mechanical cycles (Baley, 2002)**.

Plant fibers show a specific behavior under mechanical cycles. Baley (2002) was the first to show that the Young's modulus of flax fibers increases with increasing the number of cycles (Figure 3). Moreover, a plastic deformation appears after the first cycle. To explain these results, the hypothesis of a new arrangement of the microfibrils in the fiber with an increase of the crystallinity degree has been proposed by the author. Then, the more the microfibrils are aligned with the fiber axis, the better will be the mechanical properties in this direction.

A similar effect has been shown by Placet et al. (2012b) for hydrated hemp fibers. Crystallization of plant fibers under tensile test has been highlighted by Astley and Donald (2003) using the X-ray diffraction. Reorientation of microfibrils during the tensile test has also been confirmed by various studies (Keckes et al., 2003; Burgert, 2006; Placet et al., 2011).

The mechanical performance of plant fibers are influenced by different parameters including: the cellulose content, the microfibrillar angle, the fiber diameter, the temperature, the presence of defects and the water content inside fibers. The latter case will be the purpose of a next section.

As cellulose is the stiffer component of natural fibers, the higher the cellulose content is, the better will be the mechanical properties. The microfibrillar angle has also a major influence on the elastic properties of the plant fibers. In fact, the weaker is this angle, better are the properties because plant fibers behave as a composite material which presents better mechanical properties in the reinforcement direction. Regarding the influence of diameter, most studies conducted on plant fibers in traction showed that both the Young's modulus and tensile strength increased when the diameter of the tested fibers decreased (Baley, 2002; Andersons et al., 2005; Charlet et al., 2010; Duval et al., 2011). Recently, Placet et al. (2012a) found out the causes of this dependence while studying hemp fibers. Using a mathematical model and reconstructing a 3D image of the fibers, they showed that their Young's modulus is dependent primarily on the size of the lumen and secondly on the diameter of the fiber outer layer. The temperature also affects significantly the mechanical properties of such fibers. It can lead to the emergence of defects resulting in a decrease of the overall mechanical properties of the fibers (Gassan et al., 2001; Stamboulis et al., 2001). The occurrence of defects in such materials is also a source of variability of the plant fibers mechanical properties. These defects can appear during the different extraction and processing steps of the fibers and especially during the stage of retting (Bourmaud, 2011).The influence of all these parameters has been studied in details by Mukherjee and Satyanarayanna (1986).

### Scientific obstacles to their efficient use as reinforcement in composite materials (research topics on natural fibers)

Table 2 summarizes the advantages and drawbacks of these fibers. In fact, nowadays, there are some issues that prevent their use at a large scale, in composite materials. These different points constitute interesting research works.

**Table 2:** **Advantages and drawbacks of plant fibers**.

**Advantages**	**Disadvantages**
Low cost	Hydrophilic behavior
Recyclable	Dimensional instability
Zero fingerprint CO2	Low thermal resistance
← biodegradability →	
Renewable resources	Variability
Low density	Anisotropic behavior
High specific mechanical properties	Discontinuous
Good thermal and acoustics isolation	
Non abrasive	

One of the main disadvantages related to the use of natural fibers as reinforcement in composites is the poor adhesion between fiber and matrix. In composites, the matrix acts as a binder to transfer fibers stiffness in the material. If its adhesion with the fibers is weak, the composite will not have desired properties. In addition, it will be vulnerable to the environment in which it will be used and its lifetime should be shortened. A lot of researches are conducted to improve the adhesion of the fibers with polymeric matrix by modifying the fiber surface. Two approaches are proposed by the authors: physical treatments (plasma, corona treatment…) or chemical modification (maleic anhydride, organosilanes, isocyanates, sodium hydroxide, permanganate, and peroxide…) (Gauthier et al., 1998; Hill et al., 1998; Gassan and Bledzki, 1999; Tripathy et al., 1999; Mishra et al., 2000; Mohanty et al., 2000; Bessadok et al., 2007; Islam et al., 2010; Alix et al., 2011, 2012). Unfortunately, the treatments proposed in the literature don't always make it possible to keep the integrity of the fibers, as well as their natural character.

Another disadvantage of such fibers is the variability of their properties depending on the batch, the variety and even the location of the fiber in the plant. For example, comparing the mechanical properties of flax fibers, located at different positions in the stem (Charlet et al., 2007) showed that flax fibers located in the center had better mechanical properties than the others.

The low temperature resistance of these fibers constitutes another drawback. Thus, the process temperature of the composite in which they are fitted should not exceed 200°C. Beyond this temperature, the fiber integrity is not guaranteed. The use of natural fibers implies a restriction about the choice of the matrix.

The resistance of such fibers to fungus can also raise some problems (storage conditions, process conditions, use in humid conditions).

Finally, the hydrophilic nature of fibers is a major problem for their use as reinforcement in polymers. In fact, it has been showed that the absorption of water by the plant fibers results in a decrease of the composite performances in which they play the role of reinforcement (Rangaraj and Smith, 2000). Research has to be carried out to understand absorption mechanisms in such fibers. The following section is a literature review about their hydrophilic behavior as well as the influence of water on their properties.

## Hydrophilic behaviors of natural fibers

**Figure 4:**
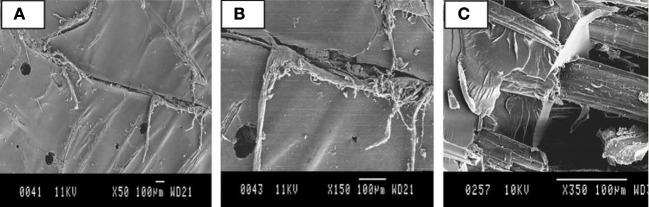
**(A)** Matrix cracking, **(B)** Fracture running along the interface, **(C)** Fiber/matrix debonding due to attack by water molecules (Dhakal et al., 2007).

For their use as reinforcement, the hydrophilic nature of plant fibers has to be considered with carefulness for several reasons. First, during the life cycle of the material, water absorption could induce a volume change of the fibers inside the composite, leading to the development of internal stresses. On the other hand, during the polymerization process of the matrix above 100°C, a vaporization of water trapped inside fibers could occur, leading to their shrinkage. These swelling and shrinkage of the fibers surrounded by the matrix generate internal stresses at the fiber/matrix interface and can eventually lead to the damage of the latter and to a significant degradation of the initial properties of the composite. The works of Rangaraj and Smith (2000); Dhakal et al. (2007); Le Duigou et al. (2009); Hu et al. (2010); Assarar et al. (2011) deal with water sorption of composites reinforced by bio-based fibers. For example, in their work on the water uptake of a flax fiber composite material (Assarar et al., 2011) showed an increase of the water content absorbed, compared to a material consisting of the same matrix reinforced with glass fibers. Le Duigou et al. (2009) studied the behavior of a composite PLLA/flax in immersion in seawater. The weight gain curves showed the influence of the cellulose fibers. The saturated moisture contents of the specimens were around 5.6%. As a consequence, adding flax fibers has resulted in a 17-fold increase in weight gain compared to unreinforced PLLA. The apparent diffusion coefficient was also significantly higher. Similar results were advanced by Lee and Wang (2006); Chow et al. (2007); Alix et al. (2011). Secondly, Le Duigou et al. (2009) showed a loss of the mechanical properties of the composite and an irreversible damage of the fiber/matrix interface during wet ageing. As mentioned before, this interface is a critical area considering the moisture absorption. The water diffusing in the composite material creates hydrogen bonds with the fibers, which can lead to the reduction of the interactions between the fibers and the matrix. Dhakal et al. (2007) showed an increase in moisture absorption with the volume fraction of fiber, for composite polyester/hemp immersed in water at 25°C. The relationship between volume fraction of fiber and water content was also clearly shown by George et al. (1998). In their work, Dhakal et al. (2007) showed a loss of mechanical properties in bending with the amount of water absorbed. According to them, the moisture absorption leads to swelling of the fiber, resulting in the occurrence of micro cracks in the matrix. Then, as the composite cracks and gets damaged, capillarity and transport via micro cracks become active. The capillarity mechanism could involve the flow of water molecules along fiber/matrix interfaces as well as a process of diffusion through the bulk matrix. This could result in a debonding of the fiber and the matrix as shown on Figure 4. Eventually, those micro cracks create swelling stresses leading to the composite failure (Bismarck et al., 2002). Debonding effect and micro cracks were also observed in the case of other bio-based composites (Chow et al., 2007; Hu et al., 2010). Concerning the evolution of the stress at break of biocomposites, there are some inconsistencies in literature. On the one hand, Dhakal et al. (2007) show the tensile stress of hemp fiber reinforced unsaturated polyester composites increases about 22% after water immersion. On the other hand, Assarar et al. (2011) show a slight decrease of the tensile stress of flax/epoxy composites after immersion (about 5%). Such a trend is not completely consistent with (Le Duigou et al., 2009) works which present a large decrease of the stress at break (50%) when flax/PLLA composites were immersed in sea water.

Finally these studies highlight the following main points:

- A significant influence of the hydrophilic behavior of cellulose fibers on the maximum moisture absorption capacity of the composite they reinforce.
- An early damage of these kinds of composites, due to swelling and shrinkage effect of fibers.

In order to study the durability of such composites materials, it is thus relevant to understand water interactions occurring in plant fibers alone. In the following sections, the link between the microstructure of the fibers and their hydrophilic behavior will be studied and the influence of water on their properties will be investigated through a review of the literature.

### Link between microstructure and hydrophilic behavior

The hydrophilic behavior of plant fibers, is mainly due to two factors: their composition and their specific structure. Generally, one of the most important factors controlling the water diffusion phenomenon in polymeric materials is the molecular interaction occurring between the diffusing compound and the substrate. The diffusion phenomenon is subjected to the ability of the polymer molecular sites to establish hydrogen bonds with the water molecules. In plant fibers, components which have polar groups and thus are responsible for absorbing moisture are cellulose, hemicellulose, pectin and lignin (Berthold et al., 1998; Célino et al., 2014a).

Some authors have studied the water absorption in cellulose (Magne et al., 1947; Nelson, 1977). They showed that the water absorbed by the cellulose has very different properties from the free water. In their works, Nakamura et al. (1981) showed a significant decrease of bound water fraction in the cellulose as the crystallinity degree of the cellulose increases, by using a differential scanning calorimetry (DSC) technique. They also revealed that water molecules bind to the 3 hydroxyl of the glycosidic units of the amorphous phase while the absorption of hydroxyl sites on the crystalline phase is unpredictable. Based on these findings, it would appear that the moisture diffusion in the cellulose takes place mainly in the amorphous phase. Thus, most of the models used in the literature to describe the hygro-elastic behavior of plant fibers consider cellulose microfibrils to be 100% crystalline and then insensitive to moisture absorption (Neagu and Gamstedt, 2007; Marklund and Varna, 2009).

According to Davies and Bruce (1998), hemicelluloses which constitute the major part of the amorphous phase in plant fibers play an important role in the storage of moisture. This hypothesis is confirmed by the results of Pejic et al. (2008), who observed a significant decrease of the saturated weight gain of hemp fibers after removal of hemicellulose and lignin. In addition, Cousins (1976, 1978) showed that their mechanical properties significantly decreased with the moisture absorption.

Pectin, located in the middle lamella and the S1 layer are composed of highly polar carboxyl functions. These groups have the ability to create hydrogen bonds with polar solvents such as water. Depending on the retting rate of the fibers, their content varies (Martin et al., 2013). So, when fiber bundles are subjected to a humid environment, moisture uptake is more important than in the case of a single fiber, as the middle lamella mainly composed by pectin is a preferential area for water absorption.

Another factor determining the high level of moisture absorption in these fibers is their particular structure. These fibers are porous and have a high exchange surface. Thus, when the fiber is subjected to a humid environment, water can be stored inside the free volume of the structure. Currently, the porosity content in plant fibers is an unknown data.

To sum up, the diffusion of water is influenced by the fiber structure at different scales. At the unit fiber scale, the fiber exhibits a complex multi cell wall structure. This structure can in first approximation be assumed to behave similarly to its thicker layer S2 which usually constitutes more than 80% of the total diameter (Gorshkova et al., 2000). Actually, this layer is assumed to be a composite material with an amorphous phase (matrix) reinforced by a rigid crystalline phase (cellulose microfibrils) (Hearle, 1963). At this scale, diffusion of water would take place in the amorphous region. Besides, these regions are mainly composed by hydrophilic polymers (hemicelluloses and lignin). At the bundle scale, diffusion is privileged trough the interface between fibers. This interface is called middle lamella. According to Morvan et al. (2003) the middle lamella is principally composed of pectin where the carboxyl functions make easier the absorption of water by hydrogen bonding. The last structural factor influencing diffusion is the general porous structure of natural fibers. Water could be trapped inside pores.

### Sorption mechanisms

The precise mechanisms governing the transport of water in these fibers are still uncertain. The moisture absorption in these bio-based materials could be due to both diffusion phenomena and the effects of capillarity.

According to Bessadok et al. (2007), at high relative humidity when the water concentration exceeds a certain threshold, there is a relaxation of the existing voids in the structure, which leads to a significant swelling of the material. In fact, it seems that water enters in the fibers and breaks the secondary bonds between the macromolecules of cellulose. Then, water molecules could link to the network via hydrogen bonds resulting in a swelling of the material (Pejic et al., 2008). Hatakeyama and Hatakeyama (1998) studied the interaction of water with hydrophilic polymers. They showed that the water was more or less linked to the network of the material, highlighting the presence of bound and free water within such structures. The amount of bound water depends on the chemical structure of the material. This water is bound to the network by hydrogen bonds, breaking the existing bonds between the hydroxyl groups of the polymer chain.

**Figure 5:**
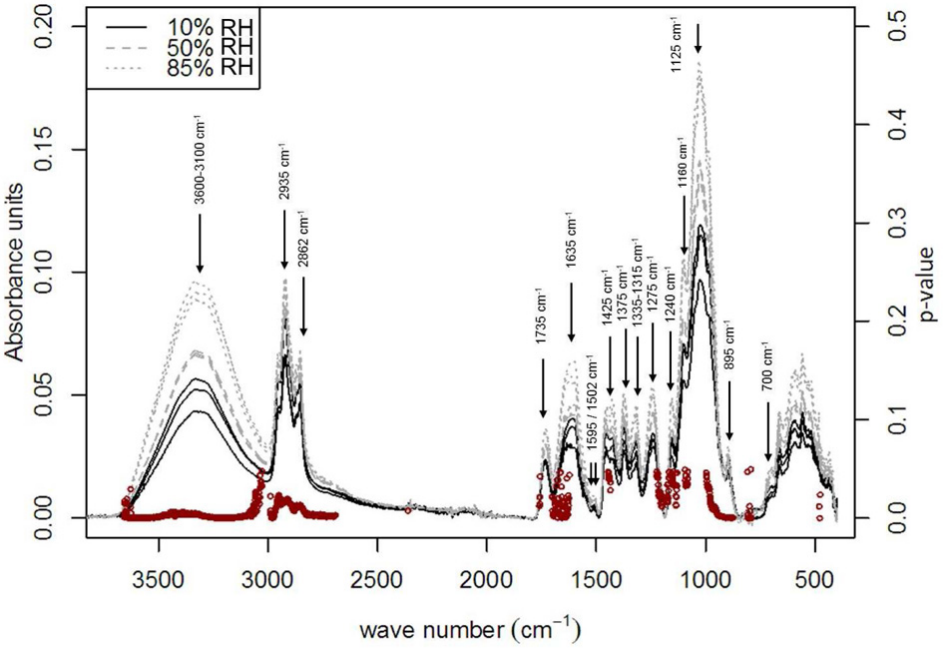
**Infrared spectra bands impacted by increasing relative humidity for sisal fiber**. *p*-values scores (used with a threshold of 0.05), indicating significant impact of the water uptake on the FTIR bands, were marked using red dots (Célino et al., 2013, 2014a,b).

Techniques for quantification and visualization of bound and free water are: DSC, Nuclear Magnetic Resonance (NMR), Raman spectroscopy and infrared spectroscopy (Hatakeyama et al., 2012). In NMR, it is possible to characterize different types of water, the molecular motion of the bound water and the water interactions with specific polymeric chain of the material in which they are inserted (Popineau et al., 2005). Using DSC, Nakamura et al. (1981) visualized and quantified these two types of water in cellulose samples. According to their works, there are actually three types of water called as follows: “freezing water,” “freezing bound water,” and “non- freezing water,” The first term refers to free water while the two others are respectively, water weakly and strongly linked to the network. The amount of “non-freezing water” is directly related to the molecular structure of the material. Fourier Transform Infra-Red spectroscopy (FTIR) has also been proved to be well adapted to study water sorption phenomenon since it allows characterizing molecular interactions involving potential sorption sites for water. In literature, FTIR spectroscopy has been widely used to study water transport in polymers and particularly to study the water sorbed into epoxy resins (Fieldson and Barbari, 1993; Cotugno et al., 2001; Feng et al., 2004). Recently Célino et al. (2014a) studied the water sorption on three natural fibers (flax, hemp, and sisal) using Fourier Transformed InfraRed spectroscopy. The spectral information enabled both qualitative and quantitative analysis of the moisture absorption mechanisms. The main chemical functions involved in the water sorption phenomenon were identified (Figure 5) and the absolute water content of the fibers was determined by using a Partial Least Square Regression (PLS-R) approach. Moreover, diffusion kinetics were plotted using this technique. More detailed analysis (by deconvolution of the OH valency band for example) could lead to the quantitative determination of the free and bond water proportions, as described in different previous works on polymer-water mixtures (Cotugno et al., 2005; Mensitieri et al., 2006).

Concerning the diffusion kinetics, most of the authors historically used a classical Fick model to represent the diffusive behavior of such fibers subjected to hydrothermal ageing (Gouanvé et al., 2007; Bessadok et al., 2009). Recently, other authors proposed using the Parallel Exponential Kinetics model (PEK) to analyze the absorption and desorption curves of different cellulose fibers (Hill and Xie, 2011; Xie et al., 2011). They suppose that the diffusion process is limited by the swelling of the material and not by the diffusion phenomenon. This model represented by a double exponential, divides the diffusion kinetics into two first-order kinetics: a slow kinetics and a rapid kinetics. The physical sense of this model has been discussed by Hill and Xie (2011). In their work, the PEK parameters for sorption have been evaluated by the authors in terms of two Kelvin–Voigt elements arranged in series. Then, the force constant in the spring of each Kelvin–Voigt elements have been determined to be the equilibrium moisture content for each process, whereas the viscosity of the dashpot is represented by the time constant for each kinetic. Indeed, the adsorbed water vapor molecules exert a pressure within the cell wall leading to a dimensional change, which is equivalent to the extension of the spring in the Kelvin–Voigt model. The spring modulus therefore defines the water content of the system at infinite time (MC1, MC2). Moreover, the rate at which water molecules are adsorbed or desorbed by the system is a function of the viscosity of the dashpot in the model. The more rapidly the matrix is able to deform, the faster the rate of water ingress or egress into or out of the cell wall.

More recently Célino et al. (2013, 2014a,b) proposed to use Langmuir theory to explain the diffusion kinetics of several fibers in immersion. In this model developed by Carter and Kibler (1978) 35 years ago, the moisture absorption can be explained quantitatively by assuming that absorbed moisture consists of both mobile and bound phases. Molecules of the mobile phase diffuse with a concentration and stress independent diffusion coefficient D_γ_, and are absorbed (become bound) with a probability per unit time γ at certain sites (for example: voids within the polymer, hydrogen bonding, and heterogeneous morphologies). Molecules are emitted from the bound phase, thereby becoming mobile, with a probability per unit time β. This model is well adapted with the structure and composition of plant fibers because it takes into account free and bound water.

**Figure 6:**
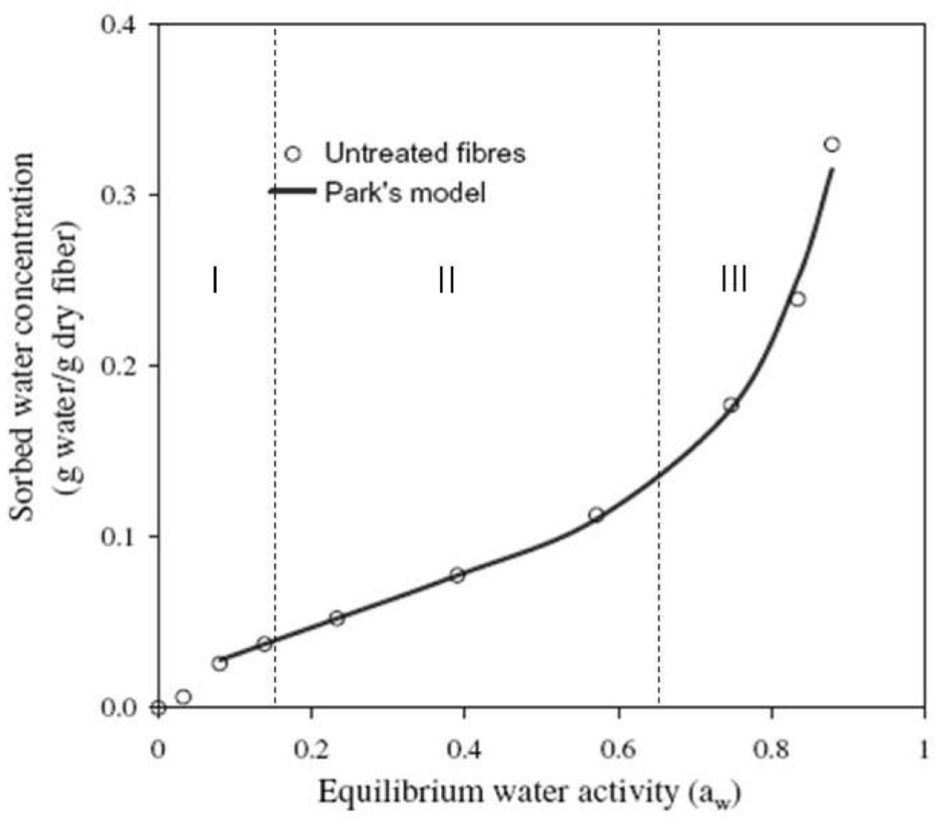
**Equilibrium water vapor sorption isotherm for modified agave fibers at 25°C [inspired by Bessadok et al. (2009)]**.

Concerning the sorption isotherms, the water content is directly related to the relative humidity by following a sigmoidal relation, as described by Alix et al. (2009); Gouanvé et al. (2007). That kind of sorption isotherms are in a good agreement with the Park's model (Park, 1986). This model assumes the association of three mechanisms describing the three parts of the curve (Figure 6) It is often used to explain the sorption isotherms of hydrophilic and porous media, as cellulosic fibers (Bessadok et al., 2009). The first part of the curve could be related to Langmuir's mode (RH < 10%). At these relative humidities, water is sorbed onto specific sites by hydrogen bonding. As previously discussed, the specific sites could be hydroxyl functions of amorphous cellulose and hemicelluloses or carboxylic function of pectin (Célino et al., 2014a). When relative humidity increases, there is a saturation of these specific sites of sorption. Then, the water concentration increases linearly with relative humidity as Henry's law describes (until RH = 65%). This behavior could be explained by the porous structure of fibers where water is free to diffuse. The third part is well described by a power function that represents an aggregation phenomenon of water molecules. Indeed, at high relative humidity, water concentration is too important, and water molecules link together to form clusters. Moreover, it has been shown that fibers immersed in distilled water at room temperature could absorb 100–200% of water by weight depending on the kind of fiber (Symington et al., 2009; Célino et al., 2013) whereas in 80% relative humidity, the water content reaches about 10–15% (Watt and Kabir, 1975; Xie et al., 2011; Célino et al., 2013). Other sorption mechanisms could explain such a gap between immersion and vapor humidity conditions.

## Effect of water on natural fibers properties

The moisture absorption in these hydrophilic fibers leads to a modification of their physical and chemical properties. Indeed, the interaction of water with hydrophilic materials may cause multiple phenomena as dimensional changes, modification of the mechanical, and chemical properties, and so on… Water can have a plasticization effect on the structure or, on the contrary, form stable hydrogen bonds leading to an anti-plasticizing effect (Hatakeyama and Hatakeyama, 1998).

### Dimensional changes

For synthetic composites, the relationship between the amount of water absorbed and the dimensional change is well documented (Weitsman, 2000). In the case of bio-composites, fibers are considered as anisotropic and hydrophilic, requiring to change models classically used for transversally isotropic and hydrophobic fibers. Quantitative information about the hydro-expansion coefficient of these fibers would be therefore an important factor for the development of new models adapted to these biomaterials. Some works have been published on the swelling of bio-composites (Madsen et al., 2012; Masoodi and Pillai, 2012). For instance, a study based on the deformation measurement of composites reinforced by hemp fibers, showed that the hydro-expansion coefficient increased with the fiber content in the material (Madsen et al., 2012). These results have also been observed by Masoodi and Pillai (2012) on a jute/epoxy bio-composite, showing that natural fibers have a strong influence on the dimensional changes of composites in which they are fitted. At the fiber scale, few study has been led to measure this coefficient despite the swelling is recognized to occur. Indeed, plant fibers have an unstable dimensional behavior. When subjected to a humid environment, they swell, resulting in the formation of internal stresses in the structure. For example, during their drying, natural fibers lose water so that shrinkage in their transverse direction could be observed. Moreover, dimensional changes of natural fibers depend on their composition. Lee et al. (2010) studied the hygroscopic deformation of cellulose microfibrils by AFM (Atomic Force Microscopy). They showed that the characteristic times of water uptake and dimensional change of the sample are not in the same scale. Indeed, there is a delay time of swelling or shrinkage of cellulose fibrils after sorption phenomenon. Hygroscopic strains may be reversible -in this case they are predicted by the hydro-expansion coefficient- or irreversible due to structural defects.

Using a hygro-elastic model applied to an elementary fiber considered as a multilayer's hollow cylinder, (Neagu and Gamstedt, 2007) highlighted the parameters influencing the hydro-expansion coefficient on wood fibers. They found that the parameter the most influential is the microfibrillar angle of the S2 layer.

### Influence on mechanical properties

Concerning the influence of water on the mechanical properties, several authors showed a relationship between moisture and mechanical properties of plant fibers. Although this influence has been clearly demonstrated, the different results of the literature are not consistent altogether (Table 3). Indeed, Davies and Bruce (1998), observed experimentally a tendency to a decrease of the Young's modulus with increasing relative humidity for flax and nettle fibers (decrease of the Young modulus of flax fibers about 23% when relative humidity varies from 30 to 80%). This trend is also highlighted by Symington et al. (2009) for flax, and Ho and Ngo (2005) for hemp and coir fibers. However, other studies show an increase of the Young's modulus of fibers with relative humidity up to a specific threshold of water absorbed (Symington et al., 2009; Placet et al., 2012b). Particularly Placet et al. (2012b) show the young modulus of hemp fibers increases about 20% in the 25–80% relative humidity range. This increase in stiffness could be explained by a rearrangement of the microfibrils and the surrounding molecules acting as a matrix (Placet et al., 2012b). This rearrangement could be activated by the swelling of the fibers. Beyond a certain moisture content threshold, the decrease of the Young's modulus could be explained by the plasticization of the fiber. In fact, the formation of hydrogen bonds replacing bonds in hemicellulose macromolecular network could make the material more flexible and compliant. Astley and Donald (2001) studied this possible realignment of microfibrils during the hydration of flax fibers using X-ray diffraction. They highlighted a structural evolution of the fibers during dehydration. Thus, they proposed a model taking into account the reorganization of microfibrils during the water molecules desorption (microfibrillar angle varying from 15° to 11° for the dry sample).

**Table 3:** **Literature review of the moisture absorption influence on the mechanical properties of plant fibers**.

**Kind of fiber**	**Hygroscopic conditions**	**Young's modulus evolution**	**Failure strength evolution**	**Elongation at break evolution**	**References**
Flax and nettle	30, 40, 50, 60, and 70%	Decreases	Not significative effect		Davies and Bruce, 1998
Flax and sisal			Maximum for RH = 70%		Van Voorn et al., 2001
Flax	30, 66, 93%		Increases and stabilizes at RH = 66%		Stamboulis et al., 2001
Hemp	10, 25, 50, et 80%	increases	Maximum for 50 < HR < 70%		Placet et al., 2012b
Jute, flax, sisal, hemp, coir, agave	65, 90% et immersion	Increases until a threshold, then decreases (depend on the fiber)	Not significative effects	increases	Symington et al., 2009

Concerning the effect of water on the maximum tensile stress, the different results of the literature are consistent. It is often observed an increase in the stress at failure with the relative humidity, up to a threshold value of RH = 50 to 60% (Placet et al., 2012b) or RH = 70% (Van Voorn et al., 2001). Above these relative humidity, the tensile strength decreases. The absorption of water inside the fiber can lead to a rupture of the hydrogen bonds between the matrix of amorphous phase and the crystalline fraction of the fiber. This would reduce the tensile strength. The literature review reveals an increase of the fiber elongation with increasing the water content. Water acts as a plasticizer and softener of the structure.

Another phenomenon, highlighted by Mannan and Robbany (1996) and more recently by Placet et al. (2012b), is the rotation of the fibers in the presence of moisture. For a static loading, the authors showed that the rotation angle increased with relative humidity. In the same work Placet et al. (2012b) observed a remarkable increase in the stiffness of the fiber during tensile tests through relative humidity cycles of RH = 50% to RH = 80%. The elastic modulus is increased by 250% from its initial value.

The diversity of these results in the literature is once again to be linked with the test conditions and variability factors of these fibers (growth conditions, extraction condition, storage condition…)

### Structural modifications

Structural modifications have been highlighted by several works. For example, in their research paper, Nakamura et al. (1983) suggest that the amorphous phase of the crystalline cellulose might become crystalline in the presence of bound water. Further tests by XRD show that the absorption of moisture in cellulose I results in an increase of the crystallinity degree. In connection with this increase of crystallinity, the authors showed an increase in tensile strength of hydrated cellulose I. The evolution of the crystalline structure of the fibers during drying was also investigated by Célino et al. (2014b) through calculating the total crystallinity index or TCI described by Nelson and O'Connor in the 60s (Nelson and O'Connor, 1964). This method supports the existence, in the cellulose infrared spectrum of both crystalline and amorphous characteristic bands. Then it is possible to estimate the fraction of the crystalline cellulose in the sample by determining the ratio of intensity of these bands. Results showed a decrease of the crystallinity degree with the decrease of the water content inside fibers, testifying the action of water on the cellulose macromolecular network. When water is removed from the sample, hydrogen bonds created between the water and the hydrophilic sites of the fibers are broken, leading to a relaxation of the macromolecular network and a decrease of the crystallinity degree. Recently, this hypothesis was confirmed by a XRD study on wood fibers submitted to hygroscopic cycles (Toba et al., 2013).

## Summary

Composites reinforced with natural fibers have developed significantly over the past years because of their biodegradability, low cost, low relative density, high specific mechanical properties, and renewable nature. These composites are predestined to find more and more applications in the near future since a lot of studies are led to understand and improve their properties. The understanding of the hygroscopic behavior of these materials is a key issue in order to use it in different weathering conditions. Many studies are examined, reviewed and highlighted in this paper regarding the link between the microstructure and the hydrophilic behavior of plant fibers, the influence of moisture on their properties as well as the final properties of the composites they reinforce. Water sorption in fibers and their composites has been found to significantly affect their dimensional and structural properties. Water sorbed in such fibers could be divided in two kinds of populations i.e., free and bound water. Free water is trapped inside the porous structure of plant fibers, whereas bound water could link to specific polar sites. These sites could be well identified by using spectroscopic techniques.

Further research is required to develop chemical or physical treatments which could reduce their water uptake. Moreover, investigations have to be conducted in order to take into account the swelling of fibers inside the composite and evaluate the internal stresses. In addition to that coupled diffusion model could be used in order to take into account the effects induced by mechanical states on the diffusion of moisture. Then, upcoming investigations could be focused on the use of more advanced multi-physics theoretical approaches dedicated to the modeling of the moisture uptake occurring while the heterogeneous, local swelling experienced by the reinforced polymer is accounted for Youssef et al. (2009, 2011); Sar et al. (2012, 2013).

### Conflict of interest statement

The authors declare that the research was conducted in the absence of any commercial or financial relationships that could be construed as a potential conflict of interest.
